# *In vitro* clustered cortical networks reveal NMDA-dependent modulation of repetitive activation sequences

**DOI:** 10.3389/fnins.2025.1650437

**Published:** 2025-09-22

**Authors:** Martina Brofiga, Mariateresa Tedesco, Francesca Bacchetti, Fabio Poggio, Bruno Burlando

**Affiliations:** ^1^Department of Informatics, Bioengineering, Robotics and Systems Engineering (DIBRIS), University of Genova, Genoa, Italy; ^2^Neurofacility, Istituto Italiano di Tecnologia (IIT), Genoa, Italy; ^3^ScreenNeuroPharm, Sanremo, Italy; ^4^Department of Pharmacy (DIFAR), University of Genova, Genoa, Italy

**Keywords:** multi-electrode array (MEA), cortical clustered networks, NMDA receptor blockade, MK-801, network plasticity

## Abstract

The development of *in vitro* networks composed of distinct but interacting neuronal sub-populations (clusters) has advanced the study of emergent behaviors in neural networks as individual functional units. In a previous work, we developed an *in vitro* model of a network formed by four mutually interconnected clusters of rat embryonic cortical neurons cultured on multi-electrode arrays (MEA), where we observed recurring, spatially and temporally structured activation sequences. In the present study, we examined the effects of NMDAR blockade (MK-801) to modulate such temporal patterns. We found that MK-801 reduced the overall excitability of the network and disrupted the diversity of repeated activation patterns, while paradoxically increasing their temporal persistence. This led the network to transition from a dynamic regime characterized by frequent and flexible repetitions to one dominated by fewer, more stable and enduring activation motifs. Functional connectivity analysis further revealed a selective weakening of inter-cluster links alongside a strengthening of intra-cluster connections. This reorganization likely explains the observed reduction in activity propagation between clusters and the simultaneous emergence of more persistent activation sequences among clusters. Data suggest that clustered neural networks serve as semi-autonomous modules, capable of sustaining internal dynamics even under diminished excitatory drive. The stable repetition of activation patterns may reflect a functional “closure” within clusters, forming self-sustained loops that enable the reactivation of previously formed motifs. From a neuroengineering perspective, this model provides a versatile platform to explore how spatiotemporal neural dynamics underpin inter-network communication, information encoding, and complex cortical functions.

## 1 Introduction

The concept that the neuron is the fundamental structural and functional unit of the nervous system has been a cornerstone of brain studies for decades. However, advances in techniques enabling the simultaneous recording of large populations of neurons have revealed that neurons can act collectively as functional units, giving rise to complex emergent properties. Building on insights from single-neuron studies, neural network models provide a promising framework for advancing our understanding of physiological processes such as balance, motor control, neuroendocrine regulation, and cognition, as well as neurological and psychiatric disorders (Yuste et al., 2024). A crucial step in studying these processes is the development of *in vitro* neural network models composed of distinct, interacting groups of neurons (Brofiga et al., 2021, 2025).

In previous work, we investigated the *in vitro* dynamics of a brain network model obtained from rat embryo cortical neurons and cultured on a multi-electrode array (MEA). The model consists of four spatially separated neuron clusters mutually interconnected by neurites (i.e., axons and dendrites), thereby forming a network of sub-networks (Brofiga et al., 2023). The data revealed rhythmic bursting activity and dominant initiating clusters, suggesting the presence of pacemaker-like behavior. Patterns of activity propagation were strongly influenced by the network’s architectural organization. The rhythmic bursting was sensitive to ivabradine, indicating a potential role for hyperpolarization-activated cyclic nucleotide-gated (HCN) channels. Activity propagated across the clusters with consistent initiator dominance and non-random activation sequences, including recurring patterns that persisted from a few seconds up to 25 s. Importantly, this effect was more pronounced in clustered neuronal networks compared to non-clustered counterparts. Collectively, these findings suggest the presence of a memory-like effect within the 4-cluster network, possibly associated with synaptic plasticity.

In the present study, we aimed to characterize the mechanism underlying the observed memory effect as an emergent property of the neural network assembly. N-methyl-D-aspartate (NMDA) receptors, which are physiologically activated by glutamate, are known to promote various forms of synaptic plasticity, including long-term (LTP) (Brown et al., 1988; Shapiro and Caramanos, 1990) and short-term potentiation (STP) (France et al., 2022). STP is prevalently presynaptic and supported indirectly by post-synaptic NMDA receptors (NMDARs) (Zucker and Regehr, 2002). The non-competitive NMDAR antagonist MK-801 is known to inhibit both short-term (Castro-Alamancos and Connors, 1997) and long-term potentiation (Gilbert and Mack, 1990), and to induce memory impairments (Janus et al., 2023). Thus, we used MK-801 to investigate the effect of NMDAR blockade in the memory-like behavior exhibited by our 4-cluster neural network model. We demonstrated that NMDAR blockade reduces overall network excitability and the diversity of repeated activation sequences, while paradoxically enhancing their temporal persistence. Functional connectivity analysis revealed a selective loss of inter-cluster connections and a strengthening of intra-cluster coupling, suggesting that network modularity supports the stabilization of repetitive motifs even under reduced excitability.

## 2 Materials and methods

### 2.1 Establishment of compartmentalized cortical networks on MEAs

Polydimethylsiloxane (PDMS) constraints were employed to structure neuronal connectivity on planar Multi-electrode Arrays (MEAs). Specifically, cross-shaped PDMS masks were used to create four physically separated neuronal assemblies, forming a 4-network system. These cross masks had equal arms measuring 2 mm in length, 0.6 mm in width, and 0.3 mm in thickness. Fabrication involved mixing PDMS prepolymer and curing agent (Sylgard 184, Sigma Aldrich) in a 10:1 (w/w) ratio, polymerized at 80 °C for 20 min. Masks were sterilized with 70% ethanol for 20 min and then reversibly aligned on MEAs to confine neuronal growth.

Primary cortical neurons were obtained from embryonic day 18–19 (E18–19) Sprague–Dawley rats. The rat embryos used for cortical neuron primary cell culture preparations were sacrificed according to the European Communities Council Directive (EU Directive 1,142,010/63/EU for animal experiments; 22 September 2010) and with the Italian D.L.No. 26/2014 and were approved by the local ethics committee and by the Italian Ministry of Health (Project Authorization No. 2018-75f11.N.POG, 2023-75F11.N.FIE). Cerebral cortices were enzymatically digested using 0.125% trypsin (Sigma Aldrich) and 0.05% DNase (Sigma Aldrich) in Hanks Balance Salt Solution (HBSS, Gibco Invitrogen) for 18 min at 37°C. The digested tissue was gently triturated, and cells were resuspended in Neurobasal medium (Gibco Invitrogen) supplemented with 2% B-27 (Gibco Invitrogen), 1% GlutaMAX (Gibco Invitrogen), and 1% PenStrep (Gibco Invitrogen).

**Figure 1:**
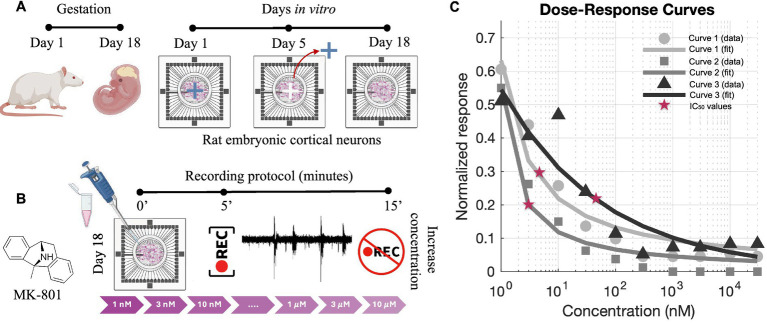
Experimental protocol and dose–response analysis for MK-801. **(A)** Schematic representation of the preparation of 4-cluster cortical networks from rat embryonic day 18 (E18) cortices. Neurons were plated on multi-electrode arrays (MEAs) and cultured. The physical barrier was removed at DIV 5 and at DIV 18 recordings were performed. During the period between the cross-shaped PDMS masks removal and the recordings, the four sub-networks developed long-range connections recreating 4-cluster neuronal networks. **(B)** MK-801, a non-competitive NMDA receptor antagonist, was delivered sequentially at increasing concentrations (1 nM to 10 μM). For each concentration, recordings were acquired after a 5-min stabilization period and lasted 10 min. **(C)** Dose–response curves generated from normalized MFR values and fitted using the Hill equation. Each curve corresponds to an independent culture preparation obtained from different animals (E18–E19 embryos), ensuring biological reproducibility of the IC₅₀ values. Red stars indicate the extracted IC₅₀ values. Panels **(A,B)** were created with BioRender.com.

Cell suspensions were plated onto pre-sterilized and poly-L-ornithine-coated (100 μg/mL) 4Q-MEAs (Multi Channel Systems) with 60 microelectrodes arranged in 4 areas. Ten microliters of the cell suspension were added to each quadrant of the cross-shaped mask at a density of 1,500 cells/mm^2^. After 2.5 h for cell adhesion, additional culture medium was added to reach a total volume of approximately 1,500 μL. Cultures were maintained at 37°C with 5% CO₂ and 95% humidity. On day 5, cross-shaped masks were removed to allow neuritic outgrowth among sub-networks (Figure 1A). The medium was then partially replaced with BrainPhys (Stemcell Technologies) supplemented with 2% NeuroCult SM1 (Stemcell Technologies), 1% GlutaMAX, and 1% PenStrep, followed by twice-weekly half-medium changes to support network maturation. More information related the polymeric structure and dissociation protocol can be found in Brofiga et al. (2023).

### 2.2 Electrophysiology and data analysis

Spontaneous electrophysiological activity from neural cultures (n = 9) was recorded at days *in vitro* (DIV) 18 using the MEA2100 system (Multi Channel Systems, MCS) at a 10 kHz sampling rate. Cultures were transferred from the incubator and allowed to stabilize for 5 min on a heated (37°C) amplifier stage before each recording session. To maintain physiological conditions, a slow, humidified gas flow (5% CO₂, 20% O₂, 75% N₂) was constantly applied above the MEA to prevent evaporation and medium acidification. Signals were acquired with MC_Rack software and processed offline using MATLAB (Mathworks, US).

#### 2.2.1 Spike and burst detection

Spikes were detected using the Precision Time Spike Detection (PTSD) algorithm (Maccione et al., 2009), which defines spikes as voltage deflections that exceed a threshold set at 8 times the noise standard deviation. Spikes had a maximum lifetime of 2 ms and a refractory period of 1 ms.

Burst detection was achieved using the string method (Chiappalone et al., 2005), identifying bursts as sequences of at least 5 spikes, with a maximum of 100 ms between consecutive spikes. Network bursts (NBs), reflecting synchronized activity across multiple electrodes, were detected using the self-adaptive algorithm (Pasquale et al., 2010) that requires a minimum percentage (20%) of electrodes involved and an inter-burst interval set at 100 ms.

#### 2.2.2 Spiking and bursting features

To quantify network activity, the following metrics were computed from the spike and burst trains: the Mean Firing Rate (MFR), i.e., the average number of spikes per second, that was also used to define “active” electrodes (MFR > 0.1 spikes/s), Mean Bursting Rate (MBR, bursts per minute), Burst Duration (BD), defined as the temporal length of the bursts, and Inter Burst Interval (IBI), i.e., the time elapsed between the end of one burst and the onset of the next one.

#### 2.2.3 Cluster activation sequences

To understand temporal dynamics during NBs, the Instantaneous Firing Rate (IFR) was calculated by smoothing spike counts using a Gaussian kernel over a 100 ms window. The IFR peak was chosen as the reference for determining the activation time of each cluster, as it corresponds to the moment of maximal coordinated activity within a cluster during a burst. This definition provides a robust marker of activation timing and avoids variability associated with threshold-based onset detection.

In the experimental set-up, physical PDMS barriers initially divided the MEA into 4 clusters (with 4 independent/not physically connected sub-networks). To evaluate the propagation of activity during NBs, each network burst was characterized by the sequence in which these clusters reached their IFR peak. These temporal patterns were termed activation sequences, starting from an “initiator cluster” (the first to activate). In order to analyze the diversity of the cluster activation sequence types, we used the Shannon diversity index (Shannon, 1948), where values close to 0 indicate low diversity (i.e., dominance of a single sequence type) and values approaching ln(N) indicate high diversity across N different sequence types. The resulting values were then normalized using the Shannon equitability index, which expresses diversity on a scale between 0 and 1 (Shannon, 1948).

#### 2.2.4 Functional connectivity analysis

To investigate functional relationships among neurons, spike trains were used to infer connectivity maps. Functional connections were identified using the Total Spiking Probability Edges algorithm, which relies on calculating the cross-correlation between spike trains of neuron pairs (De Blasi et al., 2019). To refine it and eliminate spurious connections, two thresholds were applied: one spatial (to exclude connections beyond plausible propagation distances) and one physiological (to remove edges inconsistent with synaptic timing).

#### 2.2.5 Coincidence index (CI_0_)

CI_0_ was calculated as a quantitative measure of synchrony between neuron pairs. CI₀ is defined as the ratio of the integral of the cross-correlation within ±1 ms (indicating near-simultaneous firing) to the total integral across the entire lag window. Higher CI₀ values indicate stronger functional coupling (Brofiga et al., 2021).

#### 2.2.6 Experimental protocol and dose response curves extraction

To evaluate the sensitivity of cortical networks to NMDA receptor blockade, we performed a dose–response analysis using MK-801 (Tocris Bioscience), a non-competitive antagonist of the NMDA receptor that binds within the channel pore and inhibits calcium influx in an activity-dependent manner. MK-801 was first dissolved in distilled water, further diluted in phosphate-buffered saline, and directly delivered into the culture medium at increasing concentrations. Its effect on spontaneous network activity was quantified by measuring the mean firing rate (MFR), normalized to baseline for each condition (Brofiga et al., 2023).

The experimental protocol consisted of sequential application of MK-801 at the following concentrations: 1 nM, 3 nM, 10 nM, 30 nM, 100 nM, 300 nM, 1 μM, 3 μM, and 10 μM. For each concentration, network activity was recorded after a stabilization period (5 min) to allow full drug diffusion and effect onset (Figure 1B). Normalized MFR values were fitted using the Hill equation to extract half-maximal inhibitory concentrations (IC_50_, Figure 1C). The obtained values suggested to perform experiments at the concentration of 2 nM that falls below the lowest estimated IC_50_ values (Figure 1C) and within the previously reported range (Dravid et al., 2007), ensuring only partial receptor inhibition. Importantly, this choice minimized the risk of inducing irreversible or excessively strong suppression of neuronal excitability, thus allowing us to preserve spontaneous electrophysiological activity and reliably monitor changes in activation patterns and connectivity. Following drug exposure, a washout phase was performed by removing the culture medium containing MK-801 and replacing it with pre-conditioned medium in order to avoid osmotic or metabolic shock. Recordings were acquired 5 min after medium replacement, consistent with the timing used for drug application.

#### 2.2.7 Statistical analysis

Data comparisons were carried out using: (i) the Kolmogorov–Smirnov test for cumulative frequency distributions; (ii) the Wilcoxon signed-rank test for paired samples (e.g., repeated sequence counts, functional connectivity variations); and (iii) the Student’s *t*-test with Bonferroni correction for spiking and bursting features. A significance threshold of *α* = 0.05 was applied for all tests. In the box plots, the horizontal line indicates the median, the filled circle represents the mean, and the whiskers correspond to the standard deviation. Empty diamonds denote outliers, while small dots represent the values from individual electrodes or from matrices, depending on the analysis performed.

**Figure 2:**
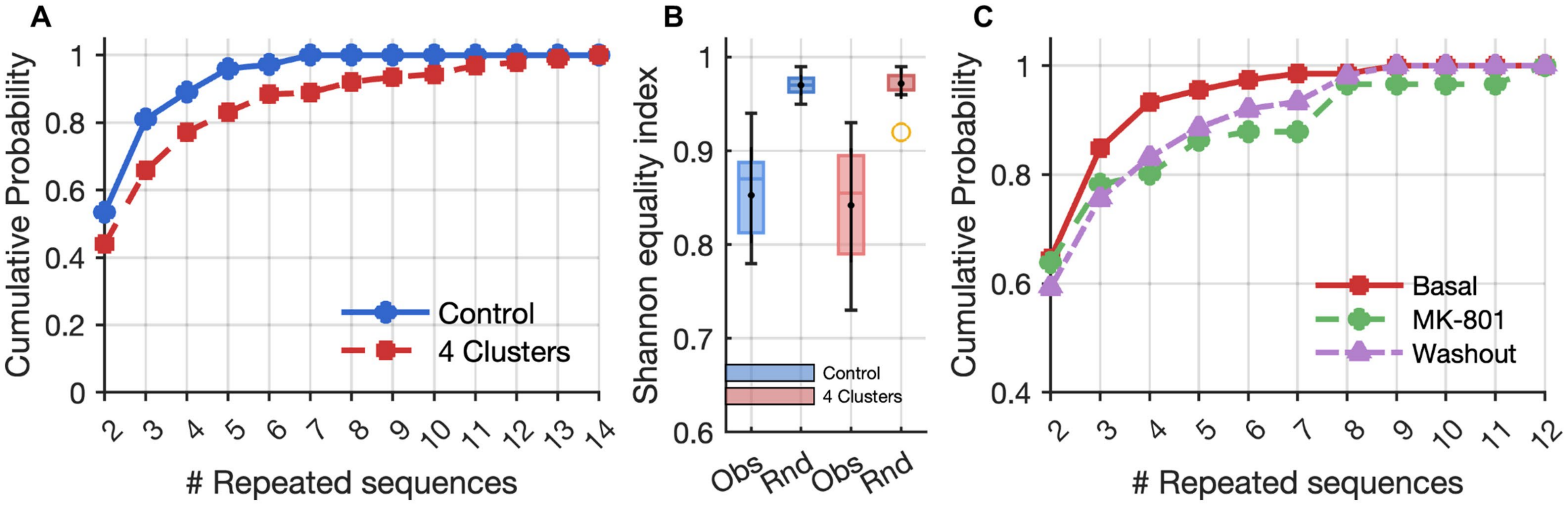
Effects of clusters and MK-801 on repeated activation sequences. **(A)** Cumulative frequency distributions based on the lengths (# Repeated sequences) of time series of identical activation sequences across neuron clusters. The control and 4-cluster arrangements showed significant differences according to the Kolmogorov–Smirnov test (*p* = 1.1 × 10^−3^). **(B)** Box plots of values representing the diversity of the activation sequences measured by the Shannon equitability index in control (blue) and clustered (red) networks. “Obs” indicates values from observed experimental sequences, while “Rnd” refers to randomized datasets (Monte Carlo simulations) used as controls. **(C)** Cumulative frequency distributions of activation sequences as in A, showing an increase in the length of time series of identical activation sequences after MK-801 treatment (*p* = 9.8 × 10^–3^, Kolmogorov–Smirnov test), and a partial recovery after washout.

**Figure 3:**
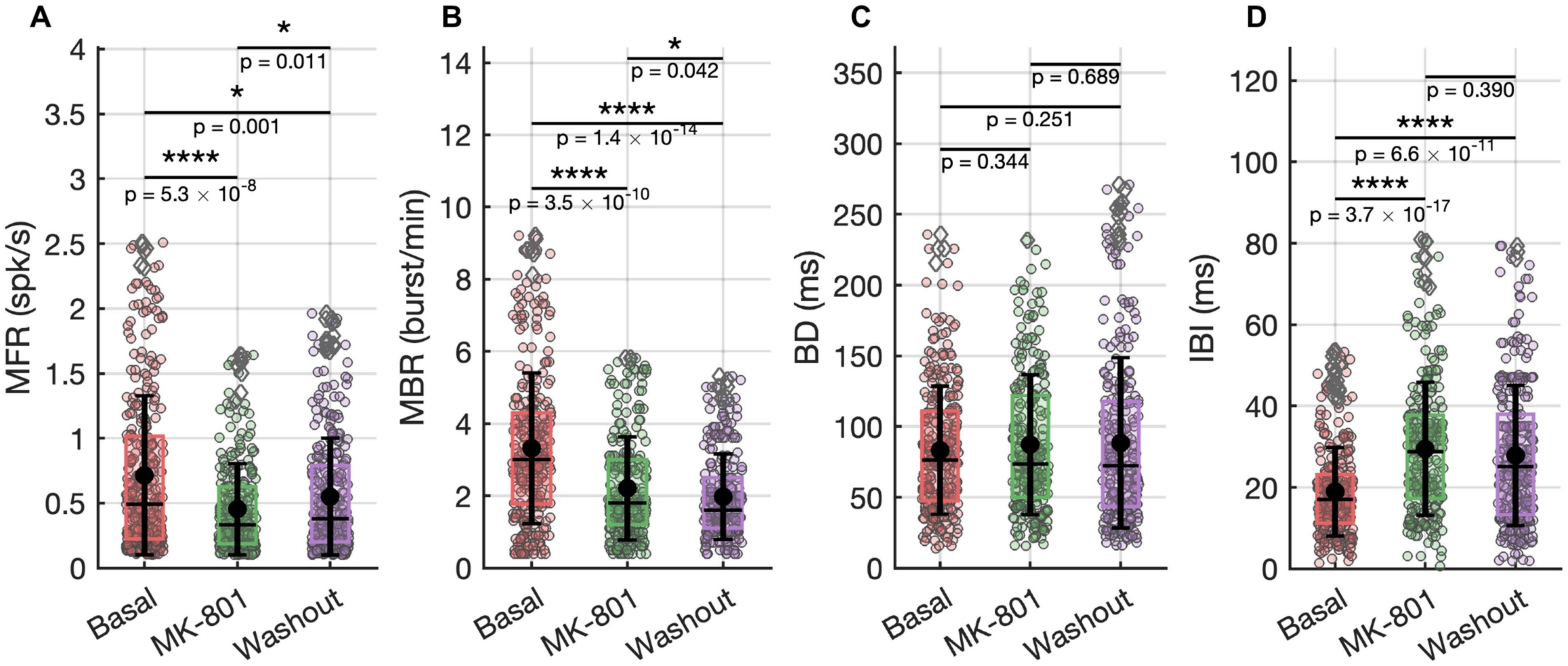
Spiking and bursting activities recorded under basal conditions, during MK-801 (2 nM) exposure, and after washout. **(A)** Mean Firing Rate (MFR). **(B)** Mean Bursting Rate (MBR). **(C)** Burst Duration (BD). **(D)** Inter Burst Intervals (IBI). Statistical comparisons were performed using a t test with Bonferroni correction.

## 3 Results

### 3.1 4-cluster networks display enhanced temporal organization compared to non-clustered network

The comparison between clustered (4-cluster) and non-clustered (control) networks revealed pronounced differences in the temporal organization of network activity, particularly in the emergence and repetition of structured activation sequences across neuron clusters. While both configurations exhibited spontaneous spiking and bursting activity, 4-cluster networks generated a significantly higher number of non-random, repeated activation sequences. Cumulative frequency analysis of repeated sequences per cluster showed a clear rightward shift for 4-cluster networks relative to the controls, indicating that spatial engineering promotes the emergence of more frequent and temporally organized repetitive motifs (Figure 2A). These patterns were characterized by both increased frequency and length of repeated activation sequences, suggesting a greater degree of functional organization and a potential substrate for memory-like behavior. Moreover, we observed a non-random set of repeated activation sequences, as evidenced by a comparison of the cumulative frequency distribution of sequence lengths between real clustered networks and Monte Carlo simulations with an equal number of sequences (Kolmogorov–Smirnov test, *n* = 421, *D* = 0.14, *p* = 0.00016, Figure 2B).

**Figure 4:**
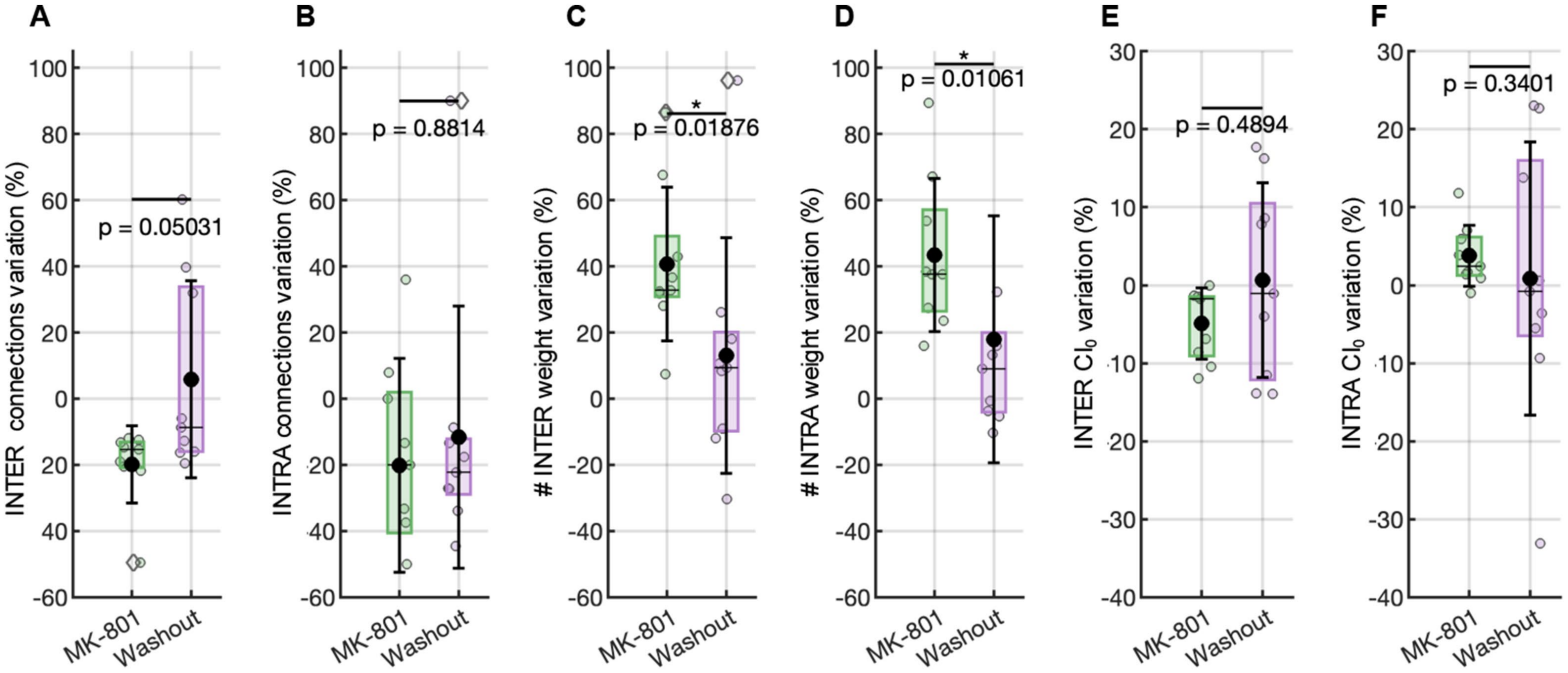
Functional connectivity evaluated with respect to the basal condition. Variation in the number of functional connections **(A)** among, and **(B)** inside the clusters. Variation in the weight connections **(C)** among, and **(D)** inside the clusters. Variation in the CI_0_ value **(E)** among, and **(F)** inside the clusters. Statistical comparisons were performed using a Wilcoxon test.

To investigate possible mechanisms underlying these emergent temporal structures and to assess their possible dependence on synaptic plasticity, we conducted a systematic analysis of network dynamics, activation sequence properties, and functional connectivity in 4-cluster networks first in baseline conditions, then during a pharmacological blockade of NMDA receptors with MK-801, and after subsequent washout (Figure 2C).

### 3.2 NMDA receptor blockade with MK-801 reduces network activity and alters repetitive sequence dynamics

To prove the role of synaptic plasticity in maintaining these temporally organized motifs, we applied MK-801, a non-competitive antagonist of NMDA receptors. A dose–response curve based on the mean firing rate was first derived (Figure 1C) to assess network sensitivity to NMDA receptor blockade. For subsequent experiments, we selected a concentration just below the estimated IC_50_ value in order to achieve a partial, yet physiologically relevant, inhibition of NMDA receptor-mediated signaling while preserving overall network viability and residual activity. This approach enabled us to examine the contribution of NMDA-dependent mechanisms to the emergence and stability of repetitive activation sequences without inducing complete network silencing.

MK-801 (2 nM) *in vitro* exposure induced a reduction in spontaneous network activity, evident across both single-neuron and population-level dynamics (Figures 3A–D). The mean firing rate (MFR) significantly decreased compared to baseline (Figure 3A), indicating a global suppression of excitability. This was paralleled by a drop in the mean bursting rate (MBR; Figure 3B), confirming impaired coordination of bursting. The inter-burst interval (IBI) significantly increased during MK-801 exposure (Figure 3C), reflecting a reduction in burst initiation frequency and prolonged silent periods between bursts. In contrast, burst duration (BD) was unaffected across conditions (Figure 3D), suggesting that the internal temporal structure of bursts remains intact despite overall suppression. These findings suggest that NMDA receptor activity could play a critical role in maintaining global excitability and regulating the timing and frequency of network-level events, while sparing the intrinsic structure of bursts once initiated.

Interestingly, the suppression of excitability was accompanied by a pronounced reorganization in the temporal structure of repetitive activation sequences. A comparison of the number of repeated sequences before and during MK-801 treatment, showed a significant decrease in the number of distinct repetitive sequences under MK-801 (Wilcoxon Signed-Rank test for paired data, n = 9, W-value = 7, *p* < 0.05; z-value = 2.09, *p* = 0.036). This finding indicates a loss of flexibility and diversity in activation motifs. However, the cumulative frequency analysis of the lengths of repeated sequence series demonstrated that, under MK-801, the curve shifted to the right, indicating that the repertoire of motifs was reduced and that the sequences which persisted tended to last significantly longer than in baseline or washout conditions (Figure 2C). This result suggests MK-801 shifted the network from a state characterized by frequent, flexible repetitions to a new one dominated by a smaller number of more stable, long-lasting activation motifs.

### 3.3 Functional connectivity remodelling under MK-801

To investigate possible alterations at the network level reflecting the temporal organization of the sequences, we examined how MK-801 impacted the structure of the functional connectivity within and between the clusters defining the networks. This analysis revealed that the observed reduction in network flexibility and increase in motif rigidity were paralleled by a distinct reconfiguration of connectivity patterns.

Specifically, during MK-801 (2 nM) treatment, the number of inter-cluster functional connections—those bridging distinct clusters—was markedly reduced (Figure 4A). This indicates that communication and integration across different modules of the network became less probable, in line with the decreased variability and propagation of activation sequences across the entire assembly. In contrast, the number of intra-cluster connections—those within the same cluster—remained stable, supporting the persistence of tightly coupled local activity even as global coordination diminished (Figure 4B). Importantly, the efficacy (weight) of the persisting functional connections increased under MK-801 for both intra-and inter-cluster links (Figures 4C,D). This last achievement suggests that, although the network became less interconnected overall (especially across clusters), the surviving functional intra-and inter-cluster connections were characterized by higher synaptic efficacy, reflecting more robust and synchronized co-activation between those neuron pairs that remained functionally linked.

In summary, the variations observed in network activity are caused by an alteration in the functional connectivity of the network: the loss of inter-cluster connections underlies the reduced flexibility and diminished propagation of activity between network compartments, while the preservation and strengthening of intra-cluster links support the emergence of longer, more stable repetitive motifs within individual clusters.

To further probe the temporal dynamics underlying the observed changes in connectivity, we evaluated network synchrony through the Coincidence Index (CI₀), which reflects the precision of co-activation between neuronal pairs. The analysis revealed a differential effect of NMDA receptor blockade on intra-and inter-cluster synchrony. Specifically, inter-cluster synchrony showed a consistent reduction relative to baseline, as indicated by negative normalized CI₀ values (Figure 4E), reflecting impaired temporal coordination across clusters. In contrast, intra-cluster synchrony increased above baseline (i.e., values greater than 0%, Figure 4F), indicating enhanced local timing precision within clusters. These results support the hypothesis that NMDA receptor signaling is essential for maintaining precise temporal alignment between spatially distinct modules, and that its inhibition disrupts long-range coordination while promoting the formation of more internally synchronized cluster-specific activity patterns.

### 3.4 Washout restores baseline activity, sequence dynamics, and connectivity

Following the removal of MK-801 and the re-establishment of physiological conditions, many features of network activity exhibited clear signs of recovery, though not all returned entirely to baseline. MFR showed an increase compared to the MK-801 condition (Figure 3A), indicating a partial restoration of neuronal excitability. Similarly, the inter-burst interval (IBI) decreased (not significantly) relative to MK-801 (Figure 3D), suggesting a reactivation of temporal coordination mechanisms for burst initiation. In contrast, the mean bursting rate (MBR) remained significantly lower than baseline (Figure 3B), pointing to a persistent disruption or delayed recovery of the network’s burst-generating capacity. In parallel with the partial recovery of firing dynamics, repetitive activation sequences also reverted toward baseline characteristics (Figure 2C). Functional connectivity analysis revealed that both the number and the strength of intra-and inter-cluster connections shifted back toward basal levels after washout of MK-801 (0% variation relative to the initial condition, Figures 4A–D), indicating a partial recovery of global connectivity architecture. Although these changes were not statistically significant, the proximity to baseline values suggests a re-equilibration of the network’s communication structure.

A similar trend was observed in the synchrony analysis: both intra-and inter-cluster CI_0_ values after MK-801 washout returned to levels near 0% change with respect to the basal condition, indicating a restoration of temporal alignment in neuronal firing patterns across and within clusters (Figures 4E,F). These findings suggest that the effects of NMDA receptor blockade on network dynamics, sequence organization, and connectivity are reversible and highlight the dynamic plasticity inherent to these neuronal assemblies.

## 4 Discussion and conclusions

Multi-cluster cortical networks display the persistence of non-random inter-cluster activation sequences, which deviated significantly from chance and pointed to an underlying form of neural plasticity (Brofiga et al., 2023). Therefore, in the present study, we sought to investigate whether this phenomenon is dependent on neuronal excitability, with a focus on the role of NMDA receptors—a key component in activity-dependent plasticity (Brown et al., 1988; Shapiro and Caramanos, 1990; France et al., 2022). To this end, we applied MK-801, a non-competitive NMDA receptor antagonist, at a concentration that reduced network excitability without abolishing spontaneous activity or the formation of activation sequences. This allowed assessing the contribution of NMDA receptor-mediated signaling to the emergence and maintenance of structured spatiotemporal activity.

Our findings align with and extend previous research demonstrating the importance of activation sequences in both *in vivo* and *in vitro* systems. Previous studies have shown that ordered spatiotemporal firing patterns underlie essential cognitive functions such as working memory, decision-making, and development (Harvey et al., 2012; Griffen et al., 2013; Buzsáki, 2015). Similar motifs have also been identified in cultured neural assemblies (Rolston et al., 2007; Wagenaar et al., 2006). Notably, these studies report that the emergence of such patterns is strongly dependent on neuronal excitability and that NMDA receptor inhibition leads to their disruption (Luhmann et al., 2016).

Consistent with these findings, we observed that 2 nM MK-801 *in vitro* exposure significantly reduced the total number of activation sequences, confirming that excitability contributes to the formation of temporally structured patterns. However, in contrast to expectations, the sequences that persisted became more temporally rigid and repetitive. Such a counterintuitive result suggests that while excitability is necessary for the generation of diverse motifs, the persistence of specific activation sequences may instead reflect a higher-order property of the network organization, rather than being solely dependent on plasticity mechanisms. Although both somatic manipulations of excitability (e.g., through sodium, calcium, or potassium channel modulation) and NMDA receptor blockade reduce firing rates, their effects on network dynamics are not equivalent. Somatic channel modulation produces a uniform reduction in spiking, whereas NMDA receptor inhibition alters synaptic integration and temporal summation, thereby directly impacting the timing and persistence of activation sequences. Analyses of functional connectivity and synchrony support this interpretation. The clustered architecture of the network promotes a non-random topological structure wherein (1) dense inter-cluster connectivity increases the likelihood of local synchronous firing; (2) inter-cluster links facilitate the ordered propagation of activity across the network. Given this combination of intra-and inter-cluster effects, the induction of a less excitable network state is likely to produce specific effects at different network functioning levels.

At the intra-cluster level, reduced spiking variability appears to enhance the detectability and strength of functional connections. With lower synaptic noise, co-active neuron pairs exhibit stronger apparent coupling, potentially reinforcing their connectivity through repeated activation. As a result, neurons within clusters may continue to strengthen their mutual connections through repeated co-activation, potentially via non-NMDA-dependent forms of plasticity.

At the inter-cluster level, the opposite pattern emerges: the number of functional connections decreases, reflecting reduced coordination between asynchronously firing subnetworks. Given that inter-cluster communication relies on precise timing, the disruption of NMDAR signaling likely impairs long-range synchronization, leading to a breakdown in inter-network integration.

This dual-level effect may explain the paradoxical persistence – and even reinforcement – of certain activation sequences under reduced excitability. When NMDA-dependent plasticity is suppressed, existing activation motifs can no longer be actively reshaped, leading to the stabilization and repeated expression of pre-existing patterns.

Taken together, these findings support a model in which clustered networks operate as semi-autonomous modules, capable of generating stable internal dynamics even under conditions of reduced excitatory drive. The observed repetition of activation sequences may thus reflect the functional “closure” of individual clusters into self-sustaining loops, enabling the re-emergence of previously established motifs. These findings are consistent with our previous observation that neuronal clustering promotes the stabilization of repeated activation sequences over time (Brofiga et al., 2023), and now extend that evidence by demonstrating the robustness and reproducibility of this phenomenon across distinct experimental conditions and manipulations. In addition to this, the unexpected enhancement of the phenomenon following a reduction in neural network excitability supports the idea that such activation sequences represent a general and fundamental feature of self-organizing neural networks.

## Data Availability

Peak trains of the dataset and Matlab functions have been deposited in Zenodo. The DOI of the deposited data and code reported in this paper is https://doi.org/10.5281/zenodo.15677872.
